# How Rheumatoid Arthritis Can Result from Provocation of the Immune System by Microorganisms and Viruses

**DOI:** 10.3389/fmicb.2016.01296

**Published:** 2016-08-17

**Authors:** Marina I. Arleevskaya, Olga A. Kravtsova, Julie Lemerle, Yves Renaudineau, Anatoly P. Tsibulkin

**Affiliations:** ^1^Central Research Laboratory, Department of Clinical Laboratory Diagnostics, Kazan State Medical AcademyKazan, Russia; ^2^Department of Biochemistry and Biotechnology, Kazan Federal UniversityKazan, Russia; ^3^Laboratory of Immunology and Immunotherapy, CHU MorvanBrest, France

**Keywords:** rheumatoid arthritis, immune system provocation, Infection, microorganisms, viruses

## Abstract

The pathogenesis of rheumatoid arthritis (RA), similar to development of a majority of inflammatory and autoimmune disorders, is largely due to an inappropriate or inadequate immune response to environmental challenges. Among these challenges, infectious agents are the undisputed leaders. Since the 1870s, an impressive list of microorganisms suspected of provoking RA has formed, and the list is still growing. Although a definite causative link between a specific infectious agent and the disease has not been established, several arguments support such a possibility. First, in the absence of a defined pathogen, the spectrum of triggering agents may include polymicrobial communities or the cumulative effect of several bacterial/viral factors. Second, the range of infectious episodes (i.e., clinical manifestations caused by pathogens) may vary in the process of RA development from preclinical to late-stage disease. Third, infectious agents might not trigger RA in all cases, but trigger it in a certain subset of the cases, or the disease onset may arise from an unfortunate combination of infections along with, for example, psychological stress and/or chronic joint tissue microtrauma. Fourth, genetic differences may have a role in the disease onset. In this review, two aspects of the problem of “microorganisms and RA” are debated. First, is there an acquired immune deficiency and, in turn, susceptibility to infections in RA patients due to the too frequent and too lengthy infections, which at last break the tolerance of self antigens? Or, second, is there a congenital deficiency in tolerance and inflammation control, which may occur even with ordinary infection frequency and duration?

## Introduction

Rheumatoid arthritis (RA) pathogenesis, in the most general sense, is largely due to an inadequate immune response in genetically predisposed individuals to environmental challenges, with bacteria and viruses (latent carrier or clinical signs of infection) being the undisputed leaders among these environmental challenges (Brooks et al., 2010; Le Dantec et al., 2015b).

The microbial concept of RA triggering agents has been discussed since the 1870s (Benedek, 2006). But, in spite of this long history, a direct role of microorganisms in the disease is controversial, while the suspected pathogen list is still growing (**Table 1**). The fact is that it is impossible to make a causal link between a specific pathogen and the disease. In light of these disappointing results in understanding RA, there are calls for even larger studies with the use of more advanced technologies (Silman and Pearson, 2002; Carty et al., 2004).

**Table 1:** Pathogens suspected of triggering rheumatoid arthritis (RA).

Bacteria and viruses	Reference
Acanthamoeba polyphagamimivirus	Shadidi et al., 2002
*Acinetobacter* spp.	Carty et al., 2004
*Alphavirus*	Leirisalo-Repo, 2005
*Bordetella* spp.	Carty et al., 2004
*Borrelia burgdorferi*	Imai et al., 2013
*Campylobacter jejuni*	Shadidi et al., 2002
*Candida albicans*	Hermann et al., 1991
*Chlamydophila* spp.	Carter et al., 2010
*Escherichia coli*	Newkirk et al., 2005
*Flavivirus*	Li et al., 2013
*Haemophilus* spp.	Carty et al., 2004
*Helicobacterpylori*	Caselli et al., 1989
*Herpesviridae*	Shadidi et al., 2002; Silman and Pearson, 2002
Human Immunedeficiency Virus	Carty et al., 2004
*Leptospira interrogans* pomona	Sutliff et al., 1953
*Mycobacterium tuberculosis*	Kim H.R. et al., 2006
*Mycoplasma arthritidis*	Phillips, 1986
*Mycoplasma fermentans*	Horowitz et al., 2000
Parvovirus	Carty et al., 2004
*Porphyromonas gingivalis*	Wu et al., 2010
*Prevotella intermedia*	Martinez-Martinez et al., 2009
*Proteus mirabilis*	Wilson et al., 2000
*Rubivirus*	Hart and Marmion, 1977
*Staphylococcus aureus*	Zahiri Yeganeh et al., 2015
*Streptococcus pyogenes*	Phillips, 1986
*Tannerella forsythensis*	Ogrendik, 2009
*Yersinia enterocolitica*	Maslova et al., 2004

### Several Explanations Can Be Proposed for These Conflicting Results

First, in the absence of a defined pathogen, the spectrum of microorganisms involved in triggering RA may include polymicrobial communities or the cumulative effect of several bacterial/viral factors. The simultaneous effects of various pathogens on the immune system in an RA patient may have different direct effects. It has been demonstrated that specific infections (gastrointestinal and urogenital), hypothetically associated with the changes in the gut microbiome, could diminish or increase the risk of RA (Sandberg et al., 2015).

Second, the spectrum of bacteria and viruses, as well as the reaction of the immune system to such pathogens, with or without clinical manifestations of infection, may vary from preclinical to late-stage RA: such that any of numerous pathogens may trigger RA at a preclinical stage, but subsequently lose their influence in the advanced stage (Arleevskaya et al., 2014). Moreover, the delicate balance between pathogens and the anti-infection immune response of an RA patient may be disturbed by several factors including the limited mobility of the patients as well as the drug therapy selected.

Third, infections might not trigger RA in all cases, but only in a subset of the cases. For example, co-occurence with psychological stress and/or chronic joint tissue microtrauma might be needed. Indeed, in Swedish and Finish cohorts only 16–20% of the patients reported infection in the early phase of RA (Leirisalo-Repo, 2005); whereas in a Russian cohort this index was as high as 75% (Arleevskaya et al., 2014). The age at disease onset also seems to be important since younger patients attributed their RA to a previous infection more often than did older patients. (Soderlin et al., 2011).

Fourth, the differences in trigger factors might reflect variations in the pathogenetic mechanisms. Indeed, RA is recognized as being a multigene disorder with a huge number of genetic polymorphisms contributing to the disease pathogenesis. The differences in trigger factors may also be due to geographic, life style, drug or chemical exposures, and ethnic differences (Konsta et al., 2015; Le Dantec et al., 2015a; Lemerle et al., 2016). The diagnostic category of RA includes multiple subtypes of the disease and the different phenotypes of RA might be due to the different genotypes. In fact, the set of clinical signs, which has been known since 1782 (Jonsson and Helgason, 1996), and which we call “RA” may be the outcome of a variety of pathogenic pathways, or in other words, it is a syndrome, i.e., a group of symptoms that arise as a consequence of a number of different diseases (Weyand et al., 1998).

Accordingly, two aspects of the problem of “pathogens and RA” *versus* “RA and infections” are still debated. Is there an acquired immune deficiency in RA patients caused by too frequent and prolonged infections, which break tolerance of self-antigens? Or, is there a congenital deficiency of the tolerance and inflammation control, which may occur even with ordinary infection frequency and duration?

## Arguments for the Acquired Versus Innate Hypothesis

The results of various studies testing the acquired or the genetically determined predisposition to infections in RA are rather contradictory. These differences can be explained by several reasons. First, the conflicting results may be explained in part by features of the patient groups studied, including the particular therapeutic approach used and the specific types of infections tracked by the authors (Vandenbroucke et al., 1987; Widdifield et al., 2013; Sandberg et al., 2015).

Second, evolution of the immune system reaction to pathogens during RA development is usually not taken into account. Our 10-year follow-up showed that both early stage RA patients and their relatives suffer from more frequent and prolonged minor infections than those individuals without autoimmune diseases in their family history (Arleevskaya et al., 2014). A gradual decrease in the frequency and duration of the infectious episodes was observed in RA patients at a later stage, when they were taken under observation at an early stage and observed for longer than 3 years. It was also observed from this cohort that the frequency and duration of the infectious episodes increased even more in the year before the RA onset, and that all the relatives who developed RA during the observation (i.e., included in the study at the pre-clinical stage) had a pronounced infectious syndrome (Arleevskaya et al., 2014). It is noteworthy that Germano et al. (2014) reported an association between the infection risk and disease activity, while these authors also supported the hypothesis that the infectious syndrome decrease with RA duration. With this in mind, we speculate that there is probably both a congenital and or an acquired deficiency of the anti-infection defense leading to the frequent and prolonged minor infections in early RA patients and their relatives. Attempts to eradicate the infections eventually lead to a certain amount of success, but at the cost of RA onset due to an inappropriate activation/inhibition of various key parts of the immune system. It should be noted, that in late stage RA, microbial colonization (including the increased frequency of heavy *Escherichia coli* and *Staphylococcus aureus* colonization) persists (Arleevskaya et al., 2014). So, in late stage RA, despite the reduction of clinical signs of frequent and prolonged infectious episodes, there are still laboratory signs of dysbiosis. Thus, a delicate balance of microflora and the immune host defense might be disrupted at any time, for example, when there is a change in the therapy. This hypothesis is indirectly confirmed by the data, indicating that a history of previous infections appeared to be among the risk factors for the infectious complications during infliximab and disease-modifying anti-rheumatic drug therapy (Widdifield et al., 2013). We interpret these data in such a way that, in RA patients with a deficiency in their anti-infection defenses, which has been manifested earlier in any infections and compensated for to some extent later, the risk of renewal of infections still remains high.

There are two possible approaches to the problem of “infections and RA”. One of them, being of particular importance for practicing rheumatologists, aims to study the susceptibility to infections as a prognostic factor for the infectious complications in RA therapy. The goal is to study all patients without exception, including those with a variety of reasons for the development of infection complications, even in the absence of RA (such as leucopenia, low mobility, diabetes, or other comorbiditiesl; Doran et al., 2002; Soderlin et al., 2011). The results of these studies indicate an increased susceptibility to infections in RA patients. The second approach is to study the role of infections in the provocation and persistence of RA activity. In this case, it seems logical (albeit with obvious damage to the completeness of coverage of the problem) not to extend the study on the patients with the concomitant additional risk factors for the infection development.

## The Acquired Hypothesis

There is a definite list of possible mechanisms by which the pathogen/host interactions could trigger pathological autoimmune responses: molecular mimicry, epitope spreading, polyclonal lymphocyte activation, bystander activation, and viral persistence (Bach, 2005; Kivity et al., 2009; Vojdani, 2014; Floreani et al., 2015).

### Molecular Mimicry

Molecular mimicry occurs when foreign antigens bear sufficient structural similarity to self-antigens (i.e., similar epitopes). As a result, an immune response to pathogens could lead to cross reactivity with self-antigens. The similarity of pathogen and self-antigen structures is a widespread phenomenon. By exact peptide matching analysis, it was demonstrated that all human proteins harbor a bacterial penta- or hexa-peptide motif (Trost et al., 2010). Moreover, the study of pathogenic and non-pathogenic proteomes from *Vibrio cholera* and *Mycobacterium tuberculosis* up to *Lactobacillus acidophilus* and *Bifidobacterium adolescentis* showed that the bacterial pathogenicity does not affect the cross-reactivity with human proteins. So, molecular mimicry would seem to be too commonplace to shift on it the blame for the development of autoimmune diseases. In the inflammatory foci a variety of bacterial proteins undergo several different types of post-translational modifications, and most of these proteins have motifs similar to those found in human polypeptides.

### Epitope Spreading

Epitope spreading is a phenomenon in which the immune T or B cell response extends beyond the original epitope. In general, epitope spreading is quite a beneficial phenomenon, contributing to the ability of the immune system to attack multiple pathogens (Powell and Black, 2001). Epitope spreading develops in the process of an ongoing immune reaction during an acute or persistent infection and following tissue destruction. Epitope spreading optimizes protection against newly encountered pathogens and assists in clearing inflammatory sites of damaged endogenous proteins of the body’s own tissues. Its success is due to the ability to quickly target new epitopes and the ability to adequately destroy pathogens and clear inflammatory sites. It appears evident that, due to RA development, abnormalities occur in the mechanisms that would otherwise constrain epitope spreading only to the diversity of foreign antigens and prevent development of an immune response to self-epitopes. A complete review of these abnormalities and self-epitopes is beyond the scope of the present discussion. We’ll briefly examine only some aspects. Epitope spreading can result from a change in a protein structure. One well-known example, protein citrullination, which is a conversion of arginine to citrulline by peptidyl arginine deaminase (PAD), could be due to development of an immune reaction against the original protein or its citrullinated form, but may also arise against other citrullinated proteins.

Currently interest in the well-established link between periodontitis and RA (Helminen-Pakkala, 1968) is going through a renaissance. This reignited interest is due to clarification of the role of citrullinated peptides as epitopes with pathogenic significance in RA, and identification of the ability of *Porphyromonas gingivalis*, the main pathogen in chronic periodontitis, to citrullinate proteins by using its own PAD (Maresz et al., 2013). Thus, this situation represents a clear example of an autoimmune disease triggered by infection *via* molecular mimicry and epitope spreading mechanisms. When activated, B lymphocytes initially producing antibodies (Ab) against the citrullinated *P. gingivalis* enolase, start producing Ab against citrullinated human enolase, and then against other citrullinated endogenous proteins (reviewed in Lundberg et al., 2010). Quirke et al. (2015) compared the incidence in RA patients of increased levels of serum Ab to arginine containing proteins (enolase, vimentin, fibrinogen), and to the citrullinated modifications according to the occurrence of lung bacterial infections. As compared to RA control patients, lung infections were associated with an elevated level of Ab to citrullinated proteins.

### Polyclonal Lymphocyte Cctivation

Microbial molecules can directly induce proliferation and differentiation of T- and B-lymphocytes regardless of their antigen specificity. In the model of murine *Trypanosoma cruzi* infection, it was shown that the number of high rate immunoglobulin secreting cells of both IgM and IgG classes increased up to 100 fold each and a variety of effector T cell activities were revealed, with both Ab and cellular immune reactions being predominantly non-specific (Arala-Chaves et al., 1992). Similar observations have been recorded in various viral, bacterial and fungal infections. The authors provided the results of their experiments, showing that as much as 95% of Ab produced in response to an infectious stimulus fail to bind to the antigens used for the immunization. The polyclonal activation normally produced excessive amounts of usually low-affinity Abs, which are directed against foreign and self-antigens. With regards to expression of the anti-immunoglobulin G Abs (also known as rheumatoid factor, RF) in human RF-positive Tg murine models, results differed according to the timing of the infection. On the one hand and following acute influenza viral infection, an abortive activation of RF-positive B cells and no increase in RF production characterized infected mice as compared to the uninfected ones (Woods et al., 2007); while, on the other hand, the same group also reported that chronic bacterial infection in the RF-positive Tg mice was associated with increased RF production, which resulted from polyclonal lymphocyte activation (Soulas et al., 2005).

In RA, peripheral blood and synovial lymphocyte polyclonal activation is well documented, although its extent is limited since hypergammaglobulinemia does not occur in these patients (Becker et al., 1990; Bucht et al., 1992; Yamamoto et al., 1992; Outschoorn et al., 1993; Brown et al., 1995). When testing the serum levels of Ab free light chains (as a biomarker of B cell activation) in a large cohort of the general population, Deng et al. (2015) reported elevated levels of the polyclonal free light chains 3–5 years before clinical onset of RA, an elevated level that remains during the follow-up of the patients. The authors pointed out that the elevation was moderate, and failed to demonstrate any correlation with mortality in the RA group.

A number of the results demonstrate that the situation with the polyclonal lymphocyte activation in RA is complicated. The data of recent studies, which tested the isotype distribution, antigen specifity and affinity of the serum and synovial fluid RFs and anti-CCP Abs as well as the expression of the inherently autoreactive idiotope 9G4 in preclinical, early and advanced stages of RA, failed to clarify whether such a process is crucial in RA development (Moyes et al., 1996; Verpoort et al., 2006, Ioan-Facsinay et al., 2008; Cambridge et al., 2014, Falkenburg et al., 2015). When analyzing these data, it looks like the anti-citrullinated Abs are produced due to the ongoing immune response to citrullinated proteins, while RFs appear later and persist due to both the polyclonal and antigen-driven activation of B lymphocytes (Renaudineau et al., 2005). Cambridge et al. (2014), speculated that “in RA, autoreactive B cell specificities escape deletion, receptor editing or anergy early in their development, ultimately giving rise to a population of B cells which can also survive entry into the mature B cell compartment in the periphery.”

Another important aspect is the imperfection of the polyclonal activation of peripheral blood B-lymphocytes from RA patients. The cell response to bacterial peptidoglycan, pokeweed mitogen, or phorbol myristate acetate stimulation was significantly reduced compared to controls (Abrahamsen et al., 1978; Pardo et al., 1984). Synovial fluid lymphocytes also proliferated poorly when stimulated polyclonally (Abrahamsen et al., 1978; Petersen, 1988). Finally, RA lymphocytes stimulated by the polyclonal B cell activator Epstein-Barr virus (EBV), produced less IgM than controls after 1 week and showed increasing IgM production between 14 and 21 days, whereas, in normal lymphocytes, IgM production decreased during this period (Irving et al., 1985).

### Bystander Activation

Autoreactive cells may be expanded and activated by non-specific means or by a combination of non-specific effects with self antigens being released and presented in an inflammatory environment created by infection (Kim B. et al., 2006). Most studies examining non-cognate T cell responses have focused on CD8 T cells, primarily in viral infection models with the stimuli being the products of infected cells, or inflammatory molecules generated by chronically virus-infected tissues or the factors produced by the cells, activated during the viral molecules interaction with toll-like receptors (Miller et al., 1997; Horwitz et al., 1998; Toubi and Shoenfeld, 2004; O’Donnell and McSorley, 2014). However, bacteria, in particular the intra-macrophage bacteria, also may be responsible for the bystander activation (Das et al., 2000; McSorley, 2014; O’Donnell and McSorley, 2014). One of the main factors provoking bystander activation is related to proinflammatory cytokines. At least in experiments studying this phenomenon, the proinflammatory cytokine cocktails or any of these cytokines are typically used for the cell stimulation (Unutmaz et al., 1994; Brennan et al., 2008). Cytokine stimulation of naive and resting memory T cells results in the proliferation of the lymphocytes and display of the effector functions of the memory T cells as measured by lymphokine synthesis and help stimulate immunoglobulin production by B cells (Unutmaz et al., 1994). At the same time, T cells activated by cytokines in the absence of T cell receptor stimulation also activate monocytes and monocyte cytokine production (Sebbag et al., 1997). Evidently these processes occur in RA pathogenesis (Sebbag et al., 1997; Brennan et al., 2008).

## The Innate Hypothesis

### Human Leukocyte Antigens (HLA)

Recent results from genome wide association studies (GWAS) support a contributory role of genetic and epigenetic factors in the development of RA (Konsta et al., 2015). The most well-studied genetic example is related to the RA associated DRB1^∗^01 and ^∗^04 alleles (shared epitopes), which are effective, when expressed, in binding citrullinated peptides, and presenting them to T cell receptors which is the optimal scenario for development of an immune response against citrullinated peptides, as well as for activation of the proinflammatory Th1 cytokine production (Tian et al., 1998; Rosloniec et al., 2002b; Auger and Roudier, 2005; Gourraud et al., 2006; Ohnishi et al., 2006). Moreover in RA patients, an increased expression of the HLA-DR molecules has been reported leading to a significant low-affinity peptide presentation and activation of autoreactive peripheral T cells (Kerlan-Candon et al., 2001; Rosloniec et al., 2002a; Hill et al., 2003; Auger and Roudier, 2005; Gourraud et al., 2006, 2007; Ohnishi et al., 2006).

With regards to associations between infections and HLA-DR peculiarities in RA, HLA-DRB1^∗^0404 was associated with a low frequency of occurence of T cells specific for EBV gp110, a replicative phase glycoprotein which is critical for the EBV infection control (Toussirot et al., 1999). While opposite to this, HLA-DRB1^∗^07, an allele associated with reduced risk of developing RA, was associated with the highest frequencies of the peripheral blood T-lymphocytes specific for gp110. Therefore, it’s not surprising that EBV DNA and EBV-encoded RNA I transcripts are present significantly more often in synovial tissue of patients positive for the shared epitope (especially HLA-DRB1^∗^0404–positive; Saal et al., 1999). When considering HLA-DRB1 alleles predisposing to the development of recurrent herpes lymphocytic meningitis and CMV reactivation, results were more contrasted with the characterization of both RA-associated non share epitopes HLA-DRB1^∗^09 and DRB1^∗^15, and RA-associated share epitopes HLA-DRB1^∗^01/^∗^04 (Du et al., 2007; Kekik et al., 2009; Kallio-Laine et al., 2010; Acar et al., 2014).

### Non-HLA Genes

**FIGURE 1:**
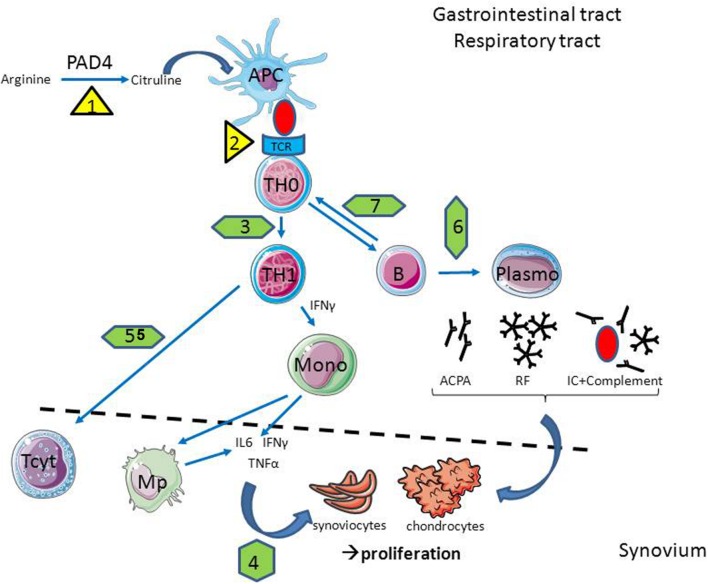
**In case of infection, the cellular and molecular players of rheumatoid arthritis (RA) are affected.** Infections are able to promote citrullinisation (1) and antigen presentation (2) through HLA-DR overexpression in antigen presenting cells (APC); to interfere with T helper (Th) polarization (3) and cytokine production (4); to reduce lymphocytotoxic activity (5) as well as antibody production (6) and antigen presentation by B cells (7). Abbreviations: Mono, Monocyte; Mp – macrophage; TCR, T cell receptor; PAD4, peptidyl arginine deaminase 4; ACPA, anti-citrullinated protein antibodies; IL, interleukine; IFNγ, interferon gamma; TNFα, tumor necrosis factor alpha; IC, immune complex; RF, rheumatoid factor.

Other gene polymorphisms associated with RA development are mainly related to imperfect control of lymphocyte activity (PTPN22, CTLA-4, BTLA, and others) (Plenge, 2009; Oki et al., 2011). Due to these SNPs the immune response becomes difficult to manage (**Figure 1**). The impaired clearance of inflammatory sites caused by an imbalance of the pro-oxidant and anti-oxidant factors and inadequate activity of several enzymes involved in remodeling of the extracellular matrix may also be affected by mutations in the genes of the corresponding factors (Mattey et al., 1999, 2000; Nemec et al., 2006; Ling et al., 2007).

### NF-κB and Jak/STAT Pathway

For all the diversity of RA-associated gene SNPs, an enrichment of these RA-associated genes was found in particular in two pathways: the NF-κB and the JAK-STAT signaling cascades (Diogo et al., 2014; Messemaker et al., 2015).

**FIGURE 2:**
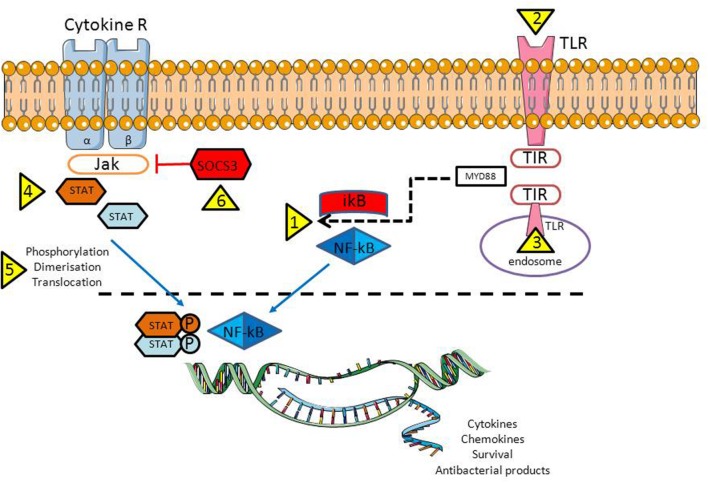
**Activation of the NF-κB and JAK-STAT signaling pathways are crucial for proper antiviral response.** However, in case of infection, bacteria/viruses have a number of opportunities to inhibit both pathways: trigger NF-κB translocation from cytoplasm into the nuclei (1) through plasma membrane (2, bacteria) or endosomal (3, viruses) TLR activation; by activing various STAT family members that being important for the virus replication (4), by controlling STAT activation/translocation to the nucleus (5); and last but not least by reducing the activity of the suppressor of cytokine signaling-3 (SOCS3), a host negative regulator of the JAK/STAT pathway (6). Abbreviations: TLR, Toll like receptor; TIR, Toll/IL-1R; MyD88, myeloid differentiation primary response gene 88; NF-κB, nuclear factor kappa B; IkB – inhibitor of the NF-κB kinase; P – phosphate residue (activated form); Cytokine R, a cytokine receptor with β1 and β2 subunits; JAK, Janus tyrosine kinase familly; STAT, signal transducer and activator of transcription family; SOCS3, suppressor of cytokine signaling 3.

The NF-κB pathway (**Figure 2**) is considered to be a prototypical proinflammatory signaling pathway controlling both RA pathogenesis and viral infection, due to the expression of proinflammatory genes of chemokines, cytokines, receptors, apoptotic regulators, intracellular signaling molecules, and transcription factors (Hinz et al., 2002; Lawrence, 2009). Indeed, stimulation of the NF-κB signaling pathway and inhibition of TNF-related apoptosis (impaired in RA even without this additional exposure) potentiate RA development. Such a pathway could be amplified in the case of infections with CMV and EBV, since the viruses need live, functional and activated “lymphocytes” which could be achieved by controlling the same NF-κB signaling pathway and, in turn, by blocking TNF-related apoptosis (Goodkin et al., 2003).

STATs are a family of proteins which are latent transcription factors activated by extracellular signaling ligands such as cytokines, growth factors and hormones (Abroun et al., 2015). The principal RA players: IL6, TNF-α as well as IFNs, exert their biological functions through the JAK/STAT signaling pathway (**Figure 2**), proteins which are being overexpressed in the various cells in RA (Rottapel, 2001; Shouda et al., 2001). STAT proteins become activated in the cytoplasm by Janus kinases (JAK), a family of tyrosine kinases. These signaling pathways have diverse biological functions, which include participating in inflammation and cell differentiation, proliferation, development and apoptosis. In particular, TNF-α production and NF-κB pathway activation can occur through JAK/STAT signaling (Ahmad et al., 2015). At the same time NF-κB positively regulates STAT5a expression and signaling pathways, and promotes persistent activation (Prosch et al., 2003).

## Lessons from Herpesviridae Infections in RA

### Herpes Simplex Virus Type 1 and 2 (HSV1/2) Prevalence and Ability to Escape the Immune System Response

Worldwide rates of either HSV-1 or HSV-2 in adults are found to be 60–95% (Chayavichitsilp et al., 2009), and due to such a high level of exposure, some authors have suggested that these viruses should be considered to be part of the normal microbiotic flora (Grinde, 2013). However, diagnosis of an HSV infection based on the presence of viral DNA in blood cells does not provide comprehensive information since HSVs can take up life-time residency in nerve cells during the latency phase and are transported to the mucosa during reactivation phases (Grinde, 2013). The latency *versus* reactivation strategy of the *Herpesviridae* is defined by the balance between viral proliferation and the ability of the immune system to clear the virus (Grinde, 2013). During the latent state, the viral genome is packaged by histones and copied by the host cell’s DNA polymerases, along with the chromosomes, primarily when the cell engages in mitosis (Knipe and Cliffe, 2008; Grinde, 2013). This contrasts with lytic replication in which the viral DNA polymerase is engaged, reflecting a viral takeover of the cell. Reactivation may be provoked by various factors, leading to activation of demethylation and histone modification processes, processes that appear to be excessive in RA (Bottini and Firestein, 2013; Wada et al., 2014, de Andres et al., 2015). As a whole, the virus’s tactics to avoid immune detection and establish latency works in a significant population – up to 80% in human adults for HSV-1 and about 40% for HSV2 (Akhtar and Shukla, 2009).

According to our data the ratio of people with clinical signs of HSV exacerbation (criteria reviewed in Grinde, 2013) is significantly increased in the early stage of RA as well as among the first degree relatives of these patients (Arleevskaya et al., 2014). However, when considering HSV1/2 DNA detection in blood, joint tissues and synovial fluid cells, an infrequent presence of HSV1/2 infection has been reported in the patients with early diseases (Zhang et al., 1993; Stahl et al., 2000). In late stage RA, the prevalence of the serum IgG anti-HSV antibodies and the viral DNA presence in the blood and synovial fluid cells were similar to that seen in controls (Us et al., 2011). The data on the serum IgM anti-HSV antibody prevalence in RA are contradictory and could not help resolve the discordance observed between the biology and clinical observations (Kurbanov and Mamedov, 2009; Us et al., 2011).

### Cellular Sensitivity and Response to Herpes Infection in RA

Since spreading of the virus from cell to cell depends on specific receptors, the presence of these receptors plays a critical role in viral infection and, in turn, in disease exacerbation (Akhtar and Shukla, 2009). Among them, the herpes virus entry mediator (HVEM), a member of the tumor necrosis factor receptor superfamily, serves as one of the entry receptors of HSV (Gavrieli et al., 2006; Cai and Freeman, 2009). In RA, HVEM is overexpressed and contributes to the proliferation and activation of synovial fibroblasts (Ishida et al., 2008). This factor is overexpressed on most cell types found in RA synovial tissues (Kang et al., 2007; Shang et al., 2012). Serum levels of soluble HVEM are increased in RA as well (Jung et al., 2003). So, HVEM overexpression in RA is suspected of contributing to HSV dissemination and to the RA progression directly or indirectly via reception of the viruses.

The epidermal growth factor receptor (EGFR) is another receptor by which HSV enters a cell. EGFR is found to be overexpressed on a variety of synovial tissue cells, and the receptor gene overexpression in bone marrow-derived mononuclear cells is due to an RA-associated SNP (Nakano et al., 2005; Nakamura et al., 2006; Lo et al., 2012; Yuan et al., 2013; Xu et al., 2015).

### Immune Surveillance of the Herpesviridae Infection in RA

**FIGURE 3:**
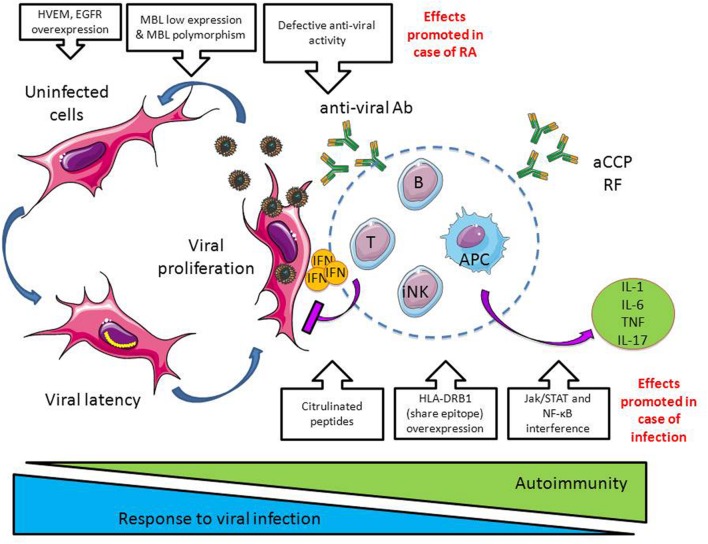
**Dangerous liaisons between infections and RA.** In case of viral infections such as observed with *Herpesviridae*, the immune system limits viral infection by producing proinflammatory cytokines (e.g., interferon, IFN), anti-viral antibodies and through the activation of cytotoxic cells such as CD8+ T cells and innate natural killer cells (iNK). In case of RA, a defective antiviral activity is observed together with factors promoting *Herpesviridae* propagation (e.g., herpes virus entry mediator, HVEM, and epidermal growth factor receptor, EGFR, overexpression; downregulation of the opsonic factor MBL, mannose binding lectin). At the opposite, infections promote autoimmunity by inducing protein citrulinisation, by promoting HLA-DR overexpression, and by interfering with the pathways involved in the production of cytokines and chemokines, Jak/STAT and NF-κB (see **Figure 2**).

The capacity of the immune system to effectively control *Herpesviridae* is determined by the adequacy of the virus (i) to directly interact with the immune system through the pattern recognition receptors (PPR), (ii) to modulate cytokine and chemokine production, and (iii) to control invariant natural killers (iNK) T cells, CD8+ cytotoxic T cells (IFN-γ, TNF-α, cytolytic molecules including perforin and granzymes), and the ability of the immune system to establish and maintain a pool of HSV-specific memory/effector CD8(+) T-lymphocytes (**Figure 3**).

#### Pattern Recognition Receptors

The viruses are recognized by PPR, among which the mannose receptors are considered to be the most important (Summerfield et al., 1995; Seppanen et al., 2009). Some preliminary evidence suggests that mannose receptors may be overexpressed on immune cells of myeloid lineage present in blood and synovial tissue from RA patients (Put et al., 2013).

One member of the secreted soluble PPR family, the mannose-binding lectin (MBL), is a complement component and an opsonic factor binding to HSV. Serum MBL levels vary according to the history of the disease. In early RA, low MBL levels were revealed, which are associated with a higher risk of developing early erosive RA and higher levels of IgM RF and CRP (Jacobsen et al., 2001; Saevarsdottir et al., 2001), and later, the MBL levels are increased (Saevarsdottir et al., 2007). Given the likely increase of MBL expression during RA evolution from early to late stage, the data by Olsen et al are of interest, showing that in the early stage of RA, the MBL gene is down regulated more than threefold compared with late stage RA (Olsen et al., 2004). The importance of MBL in RA physiopathology is reinforced by the observation of a nucleotide polymorphism (SNP) at MBL gene associated RA susceptibility (Ip et al., 2000; Jacobsen et al., 2001), and with HSV infection reccurences, possibly due to impaired recognition of the viruses (Seppanen et al., 2009).

#### Cytokines and Chemokines

The initial stages of *Herpesviridae* infection are predominantly influenced by the activity of iNK cells to produce type I interferons (IFN), which limit the spread of viruses. The other inhibitors of the infection are macrophages (producing IFNs, TNF-α, and IL-6), and lymphocytes that are instrumental in active immune surveillance by producing IFN-γ (Khanna et al., 2003; Khanna et al., 2004; Egan et al., 2013). Activated CD4+ and CD8+ T cells play a pivotal role in clearing the primary infection (Nash, 2000; Egan et al., 2013). After resolution of the primary infection, a small proportion of the primed specific T cell population generates a stable long-term memory cell pool, which activates during reactivation of a latent infection (Crough et al., 2005). Both nonspecific and specific CD8+ T cells infiltrate and persist within virus infected cells, the specific CD8+ T-lymphocytes being the prevailing ones, expressing a late effector memory phenotype and being activated by stimulation from the infected cells (Borysiewicz et al., 1988; Posavad et al., 2000; van Lint et al., 2005; Verjans et al., 2007; Zhu et al., 2007). It appears that the principal role of B cells in the immune response to *Herpesviridae* infection is not to produce neutralizing antibodies but instead to present antigens and secrete cytokines (Deshpande et al., 2000; Youinou et al., 2005, 2009; Gazeau et al., 2015).

While the IFN-γ levels were increased in the synovial fluid of early and late stage RA patients, the serum levels of the cytokines were close to that seen in controls (Bucht et al., 1996). Some authors also pointed out that, although IFN-γ was revealed in the synovial fluid and membrane, IFN-γ T cell producers were absent from peripheral blood and locally present in the joint tissues in small quantities (Ridderstad et al., 1991; De Keyser et al., 1995). T-helper 1 (Th1) cells rather than the antiviral CD8+ cytotoxic T-lymphocyte subset represent the main cellular source of IFN-γ in RA (Schuerwegh et al., 1999). Moreover, experiments using killed influenza virus (Berg et al., 2000), EBV lytic/latent peptide epitopes (Klatt et al., 2005) also support a defective capacity of the immune system to produce IFN-γ in response to viral stimulation. As a consequence, it has been proposed that a low serum level of IFN-γ may be an important factor for recurrence and reactivation of the virus (Minami et al., 2002; Davis et al., 2013; Deng et al., 2015; Motamedifar et al., 2015). A link between cytokine production and HLA alleles associated with an increased risk of RA development, HLA DRB1^∗^0401 and DQ8, has been also established (Taneja, 2015).

At the molecular level, *Herpesviridae* produce proteins that are effective to control various STAT family members and the translocation of NF-κB from the cytoplasm into the nuclei, which is necessary for (re)activation of the virus (Tsavachidou et al., 2001; Goodkin et al., 2003; Kuchipudi, 2015). As an example, STAT1 regulates the expression of the HSV-1 latency-associated transcript by interacting with its promoter (Kriesel et al., 2004). However, the STAT family proteins also regulate gene expression of a number of antiviral factors. This was elegantly demonstrated in a STAT3-deficient mouse model infected with HSV-1 that produced less IFN-γ and virus-specific CD8+ T cells (Yu et al., 2013). Another consequence of an infection by HSV-1 is its capacity to produce the suppressor of cytokine signaling-3 (SOCS3), a host negative regulator of the JAK/STAT pathway, which, in turn, inhibits the IFN-γ capacity to induce phosphorylation of JAK kinases (Yokota et al., 2004). As a whole, *Herpesviridae* have the opportunity to inhibit proinflammatory cytokine production (Melroe et al., 2004; Cox et al., 2015) through the control of the T cell response (Toussirot et al., 1999; Toussirot et al., 2000; Klatt et al., 2005). Such effect contributes to the reactivation of the *Herpesviridae* infection.

Another gene that is remarkably down regulated in early RA is the CC chemokine receptor 1 (CCR1), which regulates leukocyte migration to infection sites. Inhibition of this factor in herpes-infected mice caused a decreased and shortened recruitment of natural killer cells and led to an impaired antiviral response with a significantly higher viral level, inspite of the markedly enhanced levels of pro-inflammatory cytokines (Sorensen and Paludan, 2004). It is noteworthy that Olsen et al. (2004) concluded that, in total, the early RA signature showed some overlap with that seen in the normal immune response to viral antigens.

### Inate Natural Killers and Cytotoxic CD8 T Cells

The iNK subset is a minor population of the innate-like T-lymphocytes. They serve as an early source of cytokines (Opasawatchai and Matangkasombut, 2015). When stimulated by the altered glycolipids of the infected host cells, iNKT rapidly begin to release a variety of cytokines and chemokines, including IFNs and TNF-α, and exert direct cytolysis (Chang et al., 2007; Matsuda et al., 2008; Tessmer et al., 2009; Horst et al., 2012). An adequate response of these cells is required for control of the viral load and protection from the massive tissue damage that can occure in cases of severe viral infection (Grubor-Bauk et al., 2003; Grubor-Bauk et al., 2008). The iNK cells have specific value in keeping the virus in latency.

Several studies have unambiguously reported qualitative and quantitative abnormalities in the iNKT population in early stage RA. Mansour et al. (2015) showed that, in early stage RA, circulating iNKTs were reduced and their frequency was inversely correlated with the disease activity score. The proliferative iNKT response was also defective. Functional iNKT alterations were due to a skewed iNKT-TCR repertoire with a selective reduction of high-affinity clones. Furthermore, the high-affinity iNKTs exhibited an altered functional Th profile with Th1 (in treatment-naive) or Th2-like (in treated patients) phenotype, compared to Th0-like Th profiles exhibited by high-affinity iNKTs in controls. Cell cytotoxicity of iNK in early stage RA is also reduced (Taylor et al., 1993). It is noteworthy that in late stage RA the data, obtained by various authors were controversial: the number of the iNK and their functions were found to be increased, decreased, or didn’t differ from that seen in controls (McChesney and Bankhurst, 1986; Taylor et al., 1993; Aggarwal et al., 2014).

The population of the virus specific CD8+ T-lymphocytes and their cytotoxic activity focused on infected cells or the separate *Herpesviridae* peptide epitopes is decreased both in the early and late stage RA in contrast to an abundant and hyperactivated whole population of CD8+ T cells of various specificities (Moss et al., 1983; Gaston et al., 1986; Toussirot et al., 2000; Shimojima et al., 2008; Carvalheiro et al., 2015).

## Conclusion

Obviously, the ideal immune system response to bacterial/viral aggression should be based on the principles of a reasonable adequacy and a perfect balance between all parts of the immune system. However, for some patients such a delicate equilibrium could be broken, leading to the development of RA. Several factors are suspected for this including:

1. A susceptibility to bacterial and viral infections greater than in the general population;
2. An imbalance of the immune system greater than in the general population;
3. An incapacity to control the inflammatory reactions, and this becomes one of the major factors provoking RA development;
4. Last but not least, disharmony in the relationship of microorganisms and the immune system of an individual predisposed to developing RA, which may be due to both genetic and epigenetic problems.

## Author Contributions

All authors listed, have made substantial, direct and intellectual contribution to the work, and approved it for publication.

## Conflict of Interest Statement

The authors declare that the research was conducted in the absence of any commercial or financial relationships that could be construed as a potential conflict of interest.
